# Immune cell characteristics and cytokine responses in adult HIV-negative tuberculous meningitis: an observational cohort study

**DOI:** 10.1038/s41598-018-36696-3

**Published:** 2019-01-29

**Authors:** Arjan van Laarhoven, Sofiati Dian, Suzanne van Dorp, Feby Purnama, Valerie A. C. M. Koeken, Emira Diandini, Fitria Utami, Resvi Livia, Lika Apriani, Edwin Ardiansyah, Rob ter Horst, Mihai G. Netea, Tri Hanggono Achmad, Philip C. Hill, Rovina Ruslami, Bachti Alisjahbana, James E. Ussher, Agnes Indrati, Ayesha Verrall, Ahmad Rizal Ganiem, Reinout van Crevel

**Affiliations:** 1 Radboud University Medical Center, Department of Internal Medicine and Radboud Center of Infectious Diseases (RCI), Nijmegen, The Netherlands; 2 Universitas Padjadjaran, TB-HIV Research Center, Faculty of Medicine, Bandung, Indonesia; 3 Universitas Padjadjaran, Department of Neurology, Faculty of Medicine/Hasan Sadikin Hospital, Bandung, Indonesia; 4 Radboud University Medical Center, Department of Hematology, Nijmegen, The Netherlands; 5 Human Genomics Laboratory, Craiova University of Medicine and Pharmacy, Craiova, Romania; 6 Universitas Padjadjaran, Department of Biochemistry, Faculty of Medicine, Bandung, Indonesia; 7 Centre for International Health, Universityof Otago, Dunedin, New Zealand; 8 Universitas Padjadjaran, Department of Pharmacology and Therapy, Faculty of Medicine, Bandung, Indonesia; 9 Department of Microbiology and Immunology, University of Otago, Dunedin, New Zealand

## Abstract

Immunopathology contributes to high mortality in tuberculous meningitis (TBM) but little is known about the blood and cerebrospinal fluid (CSF) immune response. We prospectively characterised the immune response of 160 TBM suspects in an Indonesian cohort, including 67 HIV-negative probable or definite TBM cases. TBM patients presented with severe disease and 38% died in 6 months. Blood from TBM patients analysed by flow cytometry showed lower αβT and γδT cells, NK cells and MAIT cells compared to 26 pulmonary tuberculosis patients (2.4-4-fold, all p < 0.05) and 27 healthy controls (2.7-7.6-fold, p < 0.001), but higher neutrophils and classical monocytes (2.3-3.0-fold, p < 0.001). CSF leukocyte activation was higher than in blood (1.8-9-fold). CSF of TBM patients showed a predominance of αβT and NK cells, associated with better survival. Cytokine production after *ex-vivo* stimulation of whole blood showed a much broader range in TBM compared to both control groups (p < 0.001). Among TBM patients, high *ex-vivo* production of TNF-α, IL-6 and IL-10 correlated with fever, lymphocyte count and monocyte HLA-DR expression (all p < 0.05). TBM patients show a strong myeloid blood response, with a broad variation in immune function. This may influence the response to adjuvant treatment and should be considered in future trials of host-directed therapy.

## Introduction

Meningitis is the most severe manifestation of tuberculosis, leaving 30–50% of patients deceased or disabled. Immune pathology is thought to play an important role in the poor outcome of tuberculous meningitis (TBM)^1^. Adjuvant corticosteroids have shown an overall beneficial effect on survival in HIV-uninfected patients, especially in those with milder disease^2^ and are therefore part of routine care. It is conceivable, however, that a hypo-inflammatory subgroup of patients^3^ would benefit from withholding corticosteroids as currently examined in HIV-negative patients (NCT02588196), while hyper-inflammatory patients may need additional anti-inflammatory treatment^4^. More detailed information on the local and systemic immune response is needed to rationally select adjuvant agents and patient subgroups for improving host-direct therapy for TBM.

Routine cerebrospinal fluid (CSF) assessment only distinguishes mononuclear from polymorphonuclear cells. The latter, mostly neutrophils, make up on average one third of CSF cells, with higher proportions associated with a worse prognosis^5^. Microscopic study of CSF mononuclear cells has shown wide variability of cell types and counts^6^. Analysis by flow cytometry has confirmed the presence of αβT and γδT cells, B cells and Natural Killer (NK) cells in CSF of TBM patients^7,8^, but these cells have not yet been quantified. NK cells can kill extracellular *M*. *tuberculosis* and trigger effector mechanisms in macrophages^9^. Other innate lymphocyte populations might also be of importance. Mucosal associated invariant T (MAIT) cells recognize *M*. *tuberculosis* and are found in the lung in pulmonary tuberculosis^10^. NKT cell number and function are reduced in the blood of tuberculosis patients^11^. Monocytes (myeloid mononuclear cells) recognise *M*. *tuberculosis*, steer immunity and mature into macrophages with killing capacity^12^. Only one study has examined *ex-vivo* monocyte responsiveness in TBM^8^.

In this study we first characterised and quantified leukocytes in a prospective cohort of TBM patients in blood and CSF. We then established immune phenotype based on *ex-vivo* whole blood cytokine assays, using pulmonary tuberculosis patients and healthy controls for comparison. We investigated whether separate ‘high-responding’ and a ‘low-responding’ immune phenotypes exist and possibly correlate with disease phenotype and outcome.

## Methods

### Setting and patients

We prospectively included all patients >14 years of age who presented with suspected tuberculous meningitis (TBM; subacute illness including headache, fever or focal neurological symptoms) between December 2014 and July 2016 in the Hasan Sadikin hospital in Bandung, Indonesia. This is a tertiary referral hospital with 966 beds, serving the population of West-Java (43 million). Standardized screening and diagnosis as ‘definite TBM’ (CSF culture or Gene Xpert positive) or ‘probable TBM’ (CSF to blood glucose ratio was <0.5 combined with a CSF cell count ≥5 cells/μL), followed the protocols previously established in this setting^5^. Follow-up samples (day 2 and 10 for CSF and day 10, 60 and 180 for blood) were done for a subset of patients included in a clinical trial on high-dose rifampicin with inclusion up until November 2016 (NCT02169882). After hospital discharge, patients were followed-up clinically at day 90 and 180. Patients not returning to the hospital were phoned by a social worker. Cause of death, obtained from hospital records or verbal autopsy for those who died after discharge, was classified as: primarily TBM-related (i.e. brain herniation or otherwise increased intracranial pressure); pneumonia or sepsis; other, including non-infection related causes, such as injury, pulmonary embolism and aspiration pneumonia.

Pulmonary tuberculosis patients from the same hospital had chest X-ray abnormalities consistent with pulmonary tuberculosis and 25/26 patients were confirmed by positive sputum culture or smear. Asymptomatic pulmonary tuberculosis household contacts linked to the same study, who had no tuberculosis-suggestive symptoms or X-ray abnormalities and who were Interferon-γ Release Assay (IGRA)-negative were used as controls. HIV-infected patients or controls were excluded from final analysis.

### Ethics statement

Samples for this study were collected as part of three ongoing studies approved by the Ethical Committee of Hasan Sadikin Hospital/Faculty of Medicine of Universitas Padjadjaran, Bandung, Indonesia. TBM patients were included under the project “Optimization of Diagnosis of Meningitis”. This study sampled only at regular venepunctures and lumbar punctures moments that are part of routine care, for which patients gave consent orally. Consent was given by close relatives of patients who were unconscious. Consent was registered in a REDCap^13^ clinical research form for all patients. Of note, the same procedure was followed for patients over 14 and under 18 years of age, who are considered adult according to local custom. This study procedure received ethical clearance by the institutional review board of the Hasan Sadikin Hospital (Registration Number 449/UN6.C1.3.3/KEPK/PN/2015). TBM patients who were part of the high-dose rifampicin study (NCT02169882) gave separate written consent for that study (299/UN6.C2.1.2/KEPK/PN/2014). Pulmonary TB patients were part of the TANDEM study^14^ (42/UN6.C1.3.2/KEPK/PN/2015) and healthy controls were part of the INFECT study (14/UN6.C2.1.2/KEPK/PN/2014). Pulmonary TB patients and healthy controls gave written consent for sampling. All samples were handled anonymized.

### Flow cytometry

CSF was spun slowly (300 × g) for 3 minutes to avoid cell activation, the sediment was resuspended immediately (median 0.8 hours, IQR 0.7–1.0) in 2.5 mL RPMI (ThermoFisher, 22409031) and stored at 4 °C prior to flow cytometry (median 8.7 hours, IQR 4.0–16.2) later. Cell fixation interferes with measurement of activation markers and was therefore not performed. Blood was collected in lithium-heparin tubes and stored at 20–23 °C. Blood and CSF samples were processed within 24 hours of collection. Samples were divided in four equal parts, to which a fixed amount (5 μL for CSF, 30 μL for blood) of microparticles (Invitrogen 123count eBeads, 01–1234) was added for quantification, together with fluorochrome-labeled antibodies in four panels: A monocyte/neutrophil panel: CD14 (AF488), CD16 (PE), HLA-DR (PerCP) and CXCR4 (APC); a NK cell panel: CD3 (AF488), CD16 (PE), CD56 (PerCP) and CD69 (APC); a γδT/NKT cell panel: CD3 (AF488), Vα24-Jα18 (PE), Vδ2 (PerCP) and γδ-TCR (APC) and a MAIT cell panel: CD3 (AF488), Vα7.2 (PE), HLA-DR (PerCP) and CD161 (APC). Data were acquired till 1,000,000 events (blood) or a maximum of two minutes (CSF), and were analysed using Kaluza 1.3 (Beckman Coulter) using gating strategies depicted in Supplementary Fig. 1A. Flow cytometry was started in the seventh month of the study. Routine counts were measured with the Sysmex XN-1000 throughout the study.

### *Ex-vivo* whole blood stimulation assay

Stimuli were prepared in batches at 10 times the final concentration: *M*. *tuberculosis* H37Rv lysate (final concentration 5 μg/mL), *Streptococcus pneumoniae* lysate (10^6^ colony forming units per millilitre, CFU/mL), *Escherichia coli* lysate (10^6^ CFU/mL) and *Candida albicans* lysate (10^6^ CFU/mL). Live Bacillus Calmette-Guérin (BCG) was resuspended weekly from the vaccine bottle and used at 10^5^ CFU/mL. Blood obtained in lithium-heparin tubes (Greiner) was stored at 20–23 °C and processed within 24 h. Blood was diluted 1:4 with RPMI in a 24-well flat-bottom culture plate and incubated in the presence of the above mentioned stimuli, or left unstimulated, for 24 h after which supernatant was stored in two aliquots at −80 °C till batch-wise measurement by ELISA: interleukin-1 (IL-1β), IL-1 receptor antagonist (IL-1Ra), tumour necrosis factor alpha (TNF-α) (all from R&D Systems, Minneapolis, MN), and IL-6, IL-10 and interferon-gamma (IFN-γ) (Sanquin, Amsterdam, the Netherlands).

### Data analysis

Samples were excluded if they did not meet the technical minimum requirements. For flow cytometry, we excluded CSF samples obtained after start of dexamethasone or not resuspended in medium within 2.5 h, and those with inadequate fluorochrome staining or inadequate microparticle count in one of four panels. For *ex-vivo* stimulation we excluded blood samples obtained after start of dexamethasone, processed more than 24 h after acquisition, and those with TNF-α above the detection limit of 90 pg/mL for the unstimulated control. Analysis was first performed on the cytokine data for the key stimuli BCG, *M*. *tuberculosis* and *S*. *pneumoniae*, which were complete. Then, *E*. *coli* and *C*. *albicans* was included in the analysis for the patients for whom this data was complete.

Analyses were performed in R 3.2.2 (http://www.R-project.org). Non-parametric tests were used for all continuous variables. For principal component analysis (PCA), the covariance matrix was calculated on log-transformed, mean-centered, cytokine data for TBM patients using the R package prcomp. Only stimuli for which data were complete were included. Patients and controls scores were subsequently projected on the principal components (PCs) using this covariance matrix. A correlation matrix was made using Spearman correlation on pair-wise complete data. Survival analysis was performed using Cox regression with the R packages survival and visualised with survminer. Other packages used were corrplot, dplyr, openxlsx, reshape2 and tableone, and graphs were visualised using ggplot2 enhanced by cowplot.

## Results

### Patients and quality control

**Figure 1:**
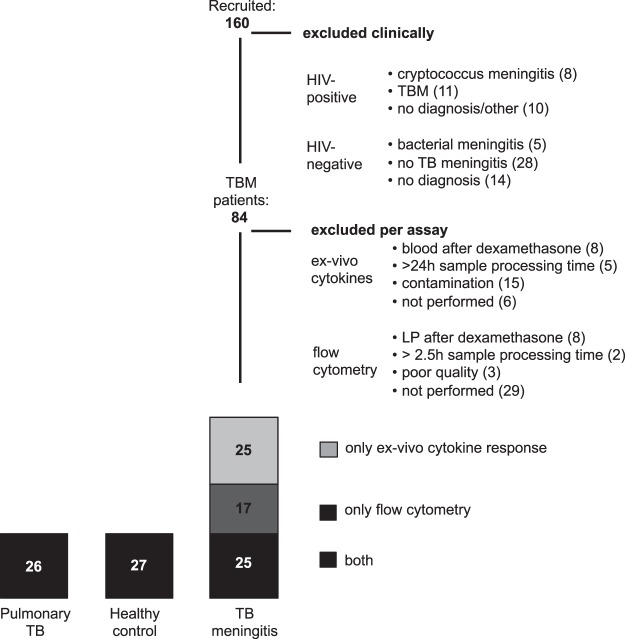
Patient inclusion diagram. Of the 26 patients with pulmonary tuberculosis, one had no *ex-vivo* cytokine response available. Of the 27 healthy controls, one had no *ex-vivo* cytokine response and one had no flow cytometry results available. LP = lumbar puncture.

Of 160 patients with suspected tuberculous meningitis (TBM) who were immunophenotyped, 47 HIV-negative patients did not meet the TBM case definition, and 29 were HIV-positive. After exclusion of samples because of technical issues or because sampling had been performed after the first dose of dexamethasone, 67 HIV-negative TBM patients could be included in the final analysis (Fig. 1). These 67 patients, 69.0% of whom had culture-confirmed TBM, presented with severe disease, 94.4% with grade II or III. TBM patients were slightly younger than pulmonary tuberculosis patients and healthy controls, while sex was similarly distributed (Table 1). Clinical follow-up was complete for all but one TBM patient and 180-day mortality was 40% (Supplementary Table 1, Supplementary Fig. 1B). Cell counts as measured by flow cytometry showed good correlation with routine cell counts (R^2^ 0.45–0.65 in blood, 0.53–0.70 in CSF, Supplementary Fig. 1C), although they were systematically lower. The ratio of cell counts using flow cytometry compared to routine counts was 0.73 for blood neutrophils, 0.76 for blood lymphocytes, 0.42 for blood monocytes, 0.19 for CSF neutrophils and 0.46 for CSF mononuclear cells. These values were not influenced by time from lumbar puncture to resuspension in medium or time to flow cytometric analysis (data not shown). Fifteen patients were excluded from *ex-vivo* cytokine analysis as result of a batch of contaminated lithium-heparin tubes.

**Table 1:** Comparison of tuberculous meningitis (TBM) patients versus pulmonary tuberculosis (PTB) patients and healthy controls (HC).

		TBM		PTB	HC	comparison (p)	
		42		26	26	TBM vs PTB	TBM vs HC
		CSF	blood	blood	blood		
sex	N-% male	23 (55%)		12 (48%)	10 (39%)	0.660	0.290
age	year	27 (20–36)		31 (24–43)	37 (24–52)	0.042	0.024
*routine haematology counts*							
total leukocytes	×10^9^ cells/L	0.216 (0.136–0.430)	10.1 (8.8–12.0)	9.1 (7.6–10.7)	6.9 (5.8–7.5)	0.088	<0.001
neutrophils	×10^9^ cells/L	0.069 (0.026–0.162)	8.6 (7.1–10.2)	6.4 (5.2–8.2)	4.0 (3.3–4.8)	<0.001	<0.001
lymphocytes	×10^9^ cells/L	0.138 (0.087–0.251)*	0.8 (0.4–1.2)	1.5 (1.3–1.8)	2.3 (1.8–2.6)	0.005	<0.001
monocytes	×10^9^ cells/L		0.7 (0.5–1.0)	0.6 (0.5–0.8)	0.4 (0.3–0.5)	0.705	<0.001
thrombocytes	×10^9^ platelets/L	n/a	267 (229–347)	396 (345–480)	285 (240–326)	<0.001	0.562
*flow cytometry results*							
total leukocytes	×10^6^ cells/L	68.4 (33.6–146.9)	7217.5 (5608.5–9258.6)	6998.1 (5129.2–8629.9)	4052.1 (3757.1–5058.8)	0.276	<0.001
CD16^+^ neutrophils	×10^6^ cells/L	12.0 (2.2–47.9)	6000.8 (4254.4–8167.5)	5011.3 (3434.9–6522.6)	2026.3 (1847.6–2874.9)	0.038	<0.001
CD16^low^ neutrophils	×10^6^ cells/L	0.5 (0.1–5.1)	113.5 (56.8–268.7)	140.7 (100.9–221.6)	237.7 (139.2–265.8)	0.684	0.101
CD14^++^CD16^−^ monocytes	×10^6^ cells/L	1.3 (0.3–3.0)	300.2 (179.7–395.1)	260.9 (181.1–353.0)	124.8 (94.3–168.2)	0.408	<0.001
CD14^++^CD16^+^ monocytes	×10^6^ cells/L	0.2 (0.1–0.5)	7.3 (2.1–15.7)	17.9 (13.0–44.8)	11.1 (5.8–18.9)	0.001	0.118
CD14^+^CD16^++^ monocytes	×10^6^ cells/L	0.1 (0.0–0.3)	1.2 (0.5–1.8)	6.9 (3.5–23.2)	10.2 (2.2–18.0)	<0.001	<0.001
CD56^-^ NK cells	×10^6^ cells/L	0.8 (0.3–2.4)	5.8 (2.5–9.4)	8.3 (5.5–20.6)	21.8 (14.7–32.7)	0.011	<0.001
CD56^+^ NK cells	×10^6^ cells/L	2.7 (1.2–7.6)	48.9 (33.7–86.5)	169.6 (94.8–198.8)	164.2 (120.9–198.1)	<0.001	<0.001
CD56^bright^ NK cells	×10^6^ cells/L	0.5 (0.2–1.3)	1.8 (0.8–3.4)	4.9 (3.2–6.9)	5.4 (3.9–7.4)	<0.001	<0.001
MAIT cells	×10^6^ cells/L	0.2 (0.1–0.8)	11.2 (7.6–30.7)	37.6 (12.2–59.4)	85.7 (37.0–134.0)	0.016	<0.001
NKT cells	×10^6^ cells/L	0.0 (0.0–0.1)	0.4 (0.1–0.8)	0.4 (0.1–1.1)	1.7 (1.3–3.4)	0.969	<0.001
Vδ2^−^ γδT cells	×10^6^ cells/L	0.2 (0.1–0.6)	6.0 (1.9–11.1)	9.0 (6.4–16.7)	15.2 (7.5–24.8)	0.014	<0.001
Vδ2^+^ γδT cells	×10^6^ cells/L	0.4 (0.1–1.5)	6.7 (3.0–15.7)	16.7 (11.4–50.3)	29.5 (14.3–60.4)	0.002	<0.001
αβT cells	×10^6^ cells/L	25.9 (17.9–44.4)	315.5 (160.7–689.7)	817.6 (705.8–1001.2)	1115.4 (950.2–1255.3)	<0.001	<0.001

Presented are the baseline characteristics of age and sex, routinely obtained cell counts and cell counts by flow cytometry. Comparisons are made by Mann-Whitney U for blood of TBM versus PTB and HC respectively.

*CSF mononuclear cells. Routine CSF leukocyte analysis by Sysmex does not differentiate lymphocytes and monocytes, together categorized as mononuclear cells.

### TBM is characterised by a predominance of myeloid cells in blood, and αβT cells in CSF

**Figure 2:**
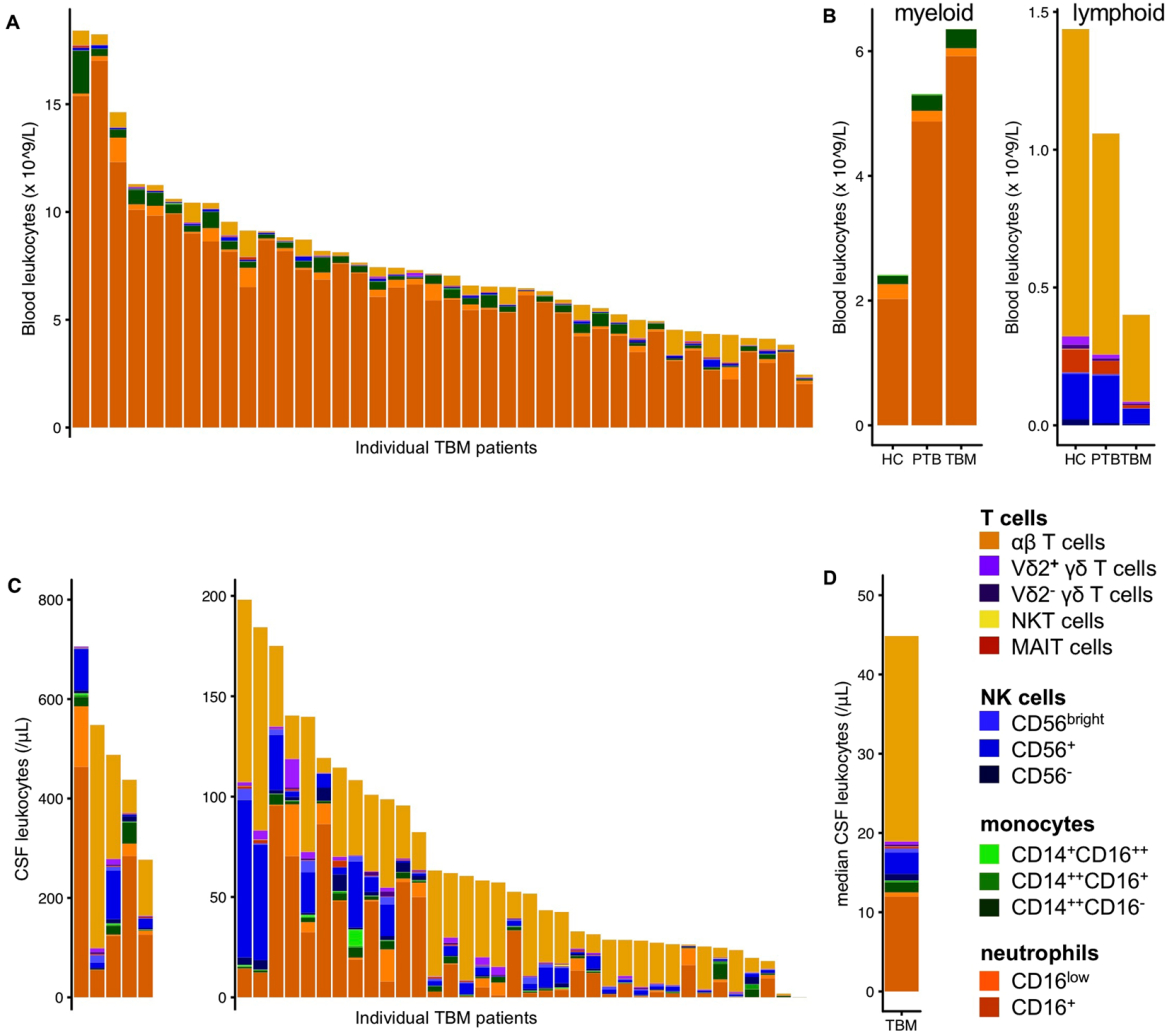
Flow cytometry results. (**A**) Blood flow cytometry results for 40 tuberculous meningitis (TBM) patients showing concentrations of individual cell types as depicted in the legend. (**B**) Median concentrations in 26 healthy controls, 26 pulmonary tuberculosis and TBM patients for myeloid (left) and lymphoid (right) cell types. (**C**) CSF flow cytometry results for 41 individual TBM patients. Patients with >200 leukocytes/μL are displayed in the left subplot and patients with ≤200 leukocytes/μL in the right subplot. (**D**) Median CSF cell composition of all TBM patients combined. Note: CD3^−^ lymphocytes without NK cell markers, most likely B cells (14%, IQR 11–43 of lymphocytes), were not included in the analysis because the flow cytometry panels lacked B-cell markers to formally confirm their phenotype.

Individual variation was large (Fig. 2A), but blood of TBM and pulmonary tuberculosis patients showed a consistently stronger myeloid response compared to healthy controls (Table 1), with increased numbers of mature (CD16^+^) neutrophils and classical (CD14^++^CD16^−^) monocytes. Intermediate (CD14^++^CD16^+^) and nonclassical (CD14^+^CD16^++^) monocytes were lower in TBM patients compared to both control groups. The lymphoid response showed the opposite: counts of all lymphocyte subsets were lower in TBM patients compared to both pulmonary tuberculosis patients and healthy controls (Fig. 2B). In patients who survived past day 10, mature neutrophils showed a further increase while the number of classical monocytes decreased. The CSF cell count of TBM patients also showed a large variation, both in total cell number (median 68/μL, IQR 34–147 as measured by flow cytometry) and in proportions of different cell populations (Fig. 2C). In the lymphoid compartment (median 49/μL, IQR 25–85), αβT cells predominated (57%, IQR 43–73 of lymphocytes), with NK cells as the second largest group (13%, IQR 9–24), with most NK cells CD56^+^. γδT cells (1.5%, IQR 0.9–3.6) were predominantly Vδ2^+^, but Vδ2^−^ cells were also present in all but one patient. Small, but well-defined populations of MAIT cells (0.4%, IQR 0.3–0.8) and NKT cells (0.06%, IQR 0.03–0.12) were found in all but one and three patients, respectively. Monocytes only made up a small proportion (2%) of all CSF cells, but this could be an underestimation because of a larger loss of myeloid cells during sample preparation. Indeed, neutrophils made up 40% of CSF cells in the routine measurement and only 17% by flow cytometry. The large majority (95%) of neutrophils were of the mature (CD16^+^) phenotype (Fig. 2D). Although variable between individuals, all blood lymphocyte subsets remained diminished during follow-up, while in CSF leukocyte counts had dropped by 59% at day 10, with αβT cells remaining the predominant cell type (Supplementary Fig. 2).

### CSF leukocytes are more activated than blood leukocytes

**Figure 3:**
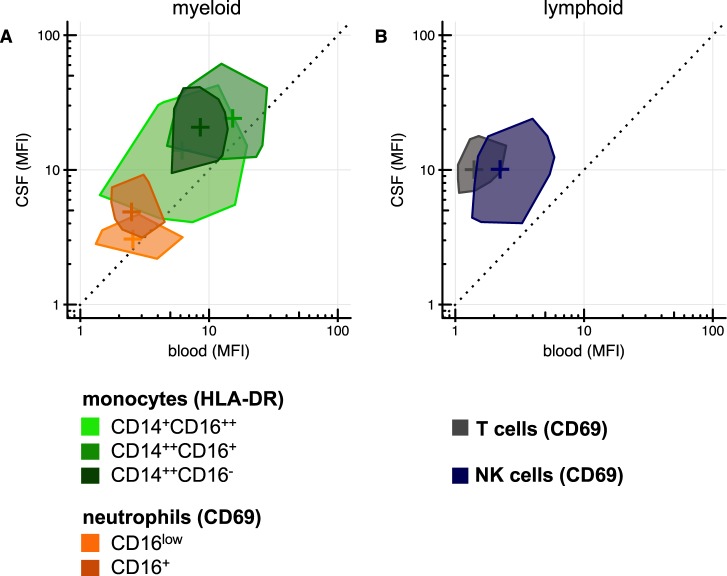
Blood versus CSF leukocyte activation. Median fluorescence intensity of activation markers in blood (x-axis) versus CSF (y-axis) for myeloid (**A**) and lymphoid (**B**) cell types. These modified ‘bag plots’ show the 50% median data-points and can thereby be compared to a two-dimension box plot without whiskers.

CD16^+^ neutrophils showed higher expression of the neutrophil activation marker CD69 in CSF compared to blood (MFI 4.3 versus 2.4, p < 0.001), while there was no difference for the smaller CD16^low^ neutrophil population. CSF monocytes showed higher HLA-DR expression in the classical (CD14^++^CD16^−^, MFI 21.3 versus 7.7, p < 0.001) and intermediate (CD14^++^CD16^+^, MFI 24.4 versus 17.7, p = 0.043) subpopulations than in blood (Fig. 3A). The difference in activation was even more pronounced in the lymphoid component, with five-fold higher CD69 on NK cells in CSF compared to blood (MFI 10.4 versus 2.0, p < 0.001) and nine-fold higher CD69 on T cells (MFI 10.8 vs. 1.2, p < 0.001, Fig. 3B). The most activated monocytes were nonclassical (CD14^+^CD16^++^) and intermediate (CD14^++^CD16^+^) monocytes that were overrepresented among CSF monocytes compared to blood. Likewise, CD56^bright^ NK cells had the largest proportional presence in CSF compared to blood among NK cells (Supplementary Fig. 3A,B). During follow-up, a gradual decrease in CSF activation was seen (Supplementary Fig. 3C).

### TBM patients show a broad range in *ex-vivo* cytokine responses

**Figure 4:**
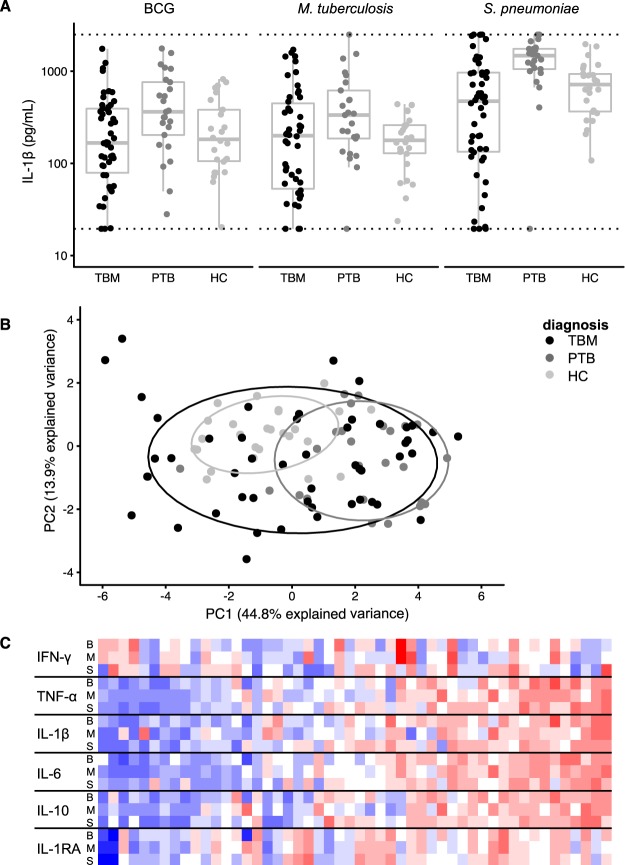
*Ex-vivo* whole blood cytokine results. (**A**) IL-1β response after stimulation of whole blood with BCG, *M*. *tuberculosis* and *S*. *pneumoniae* for each of the three patient groups tuberculous meningitis (TBM), pulmonary tuberculosis (PTB) and healthy controls (HC). (**B**) Comparison of these patient groups on principal component (PC) 1 versus PC2 in principal component analysis of all six measured cytokines for the afore-mentioned stimuli for three patient groups. (**C**) Heatmap showing the combination of six cytokines and three stimuli (y-axis; B = BCG, M = *M. tuberculosis* and S = *S. pneumoniae*) for all 50 included TBM patients (x-axis) sorted on their score on PC1.

Overall, pulmonary tuberculosis patients had the highest cytokine responses but TBM patients showed the widest range in cytokine production (example for IL-1β in Fig. 4A, Table 2). Principal component analysis on the responses to live BCG, *M*. *tuberculosis* and *S*. *pneumoniae* lysate for all six cytokines resulted in a first component (PC1, representing 45% of variation) that was largely determined by IL-1β, IL-6, IL-10 and TNF-α in response to all three stimuli (Supplementary Fig. 4A). TBM patients showed a much more heterogeneous response compared to pulmonary tuberculosis patients (p = 0.002, Levene’s test for homogeneity) and healthy controls (p < 0.001), as shown in Fig. 4B. The *ex-vivo* response of TBM patients showed a gradient in the monocyte-derived cytokines TNF-α, IL-1β, IL-6 and IL-10 after stimulation with the specific stimuli, BCG and *M*. *tuberculosis*, but also with the non-mycobacterial stimulus *S*. *pneumoniae* (Fig. 4C). Data for *C*. *albicans* (not available for every patient) showed a similar gradient while *E*. *coli* (also incomplete) did not (data not shown).

**Table 2:** Whole blood *ex-vivo* cytokine results at baseline for tuberculous meningitis (TBM) versus pulmonary tuberculosis (PTB) and healthy controls (HC).

	Patient group (n)			Comparison (p)	
	TBM	PTB	HC	TBM vs PTB	TBM vs HC
	50	25	26		
IFN-γ (pg/mL)					
BCG	18 (10–22)	21 (13–26)	16 (11–29)	0.317	0.742
*M*. *tuberculosis*	16 (12–24)	16 (12–31)	14 (8–24)	0.482	0.517
*S*. *pneumoniae*	23 (16–35)	33 (16–47)	24 (15–35)	0.070	0.622
*E*. *coli*	15 (10–19)	14 (12–18)	16 (10–26)	0.843	0.381
*C*. *albicans*	12 (9–21)	24 (17–29)	20 (15–32)	0.004	0.008
TNF-α (pg/mL)					
BCG	334 (142–847)	930 (538–1530)	252 (128–356)	0.004	0.119
*M*. *tuberculosis*	359 (97–803)	466 (215–1036)	83 (50–157)	0.214	0.001
*S*. *pneumoniae*	591 (136–1029)	1387 (1125–1668)	369 (202–647)	<0.001	0.405
*E*. *coli*	365 (104–1242)	915 (333–1569)	391 (206–773)	0.033	0.889
*C*. *albicans*	122 (52–426)	139 (70–259)	153 (100–217)	0.872	0.901
IL-1β (pg/mL)					
BCG	167 (79–393)	363 (204–761)	183 (106–382)	0.012	0.653
*M*. *tuberculosis*	200 (53–449)	335 (186–619)	178 (129–260)	0.055	0.921
*S*. *pneumoniae*	474 (134–968)	1477 (1055–1749)	721 (366–935)	<0.001	0.124
*E*. *coli*	288 (42–473)	760 (486–1037)	667 (195–944)	<0.001	0.005
*C*. *albicans*	95 (37–193)	106 (53–131)	104 (66–161)	0.713	0.499
IL-6 (pg/mL)					
BCG	2838 (1056–5108)	6052 (4477–9882)	2888 (1731–4750)	0.001	0.878
*M*. *tuberculosis*	2281 (700–6104)	4000 (2986–7269)	1574 (850–2728)	0.027	0.191
*S*. *pneumoniae*	1635 (573–3114)	2183 (1667–3047)	1530 (971–2160)	0.071	0.965
*E*. *coli*	2068 (607–3284)	3331 (2379–5149)	2179 (1363–3024)	0.009	0.901
*C*. *albicans*	1151 (411–2436)	1192 (486–1630)	694 (318–1101)	0.648	0.107
IL-10 (pg/mL)					
BCG	39 (12–81)	52 (44–84)	35 (24–53)	0.132	0.801
*M*. *tuberculosis*	21 (10–59)	36 (17–44)	14 (6–25)	0.386	0.077
*S*. *pneumoniae*	33 (15–54)	38 (21–55)	24 (13–40)	0.458	0.187
*E*. *coli*	38 (5–67)	51 (32–89)	61 (28–73)	0.106	0.258
*C*. *albicans*	6 (5–32)	5 (5–13)	6 (5–9)	0.457	0.383
IL-1RA (pg/mL)					
BCG	3810 (2020–6132)	4833 (3210–6209)	1597 (1290–2269)	0.160	<0.001
*M*. *tuberculosis*	3366 (1924–6719)	4651 (2677–5980)	1645 (1325–2067)	0.351	<0.001
*S*. *pneumoniae*	3948 (1826–5861)	6083 (4499–10546)	1728 (956–2270)	0.002	<0.001
*E*. *coli*	3902 (1647–6512)	6901 (3789–8459)	2830 (2329–3739)	0.006	0.143
*C*. *albicans*	1633 (1132–3071)	2334 (1751–3361)	908 (604–1578)	0.146	0.002

Mann-Whitney U p-values for are shown. Data were missing for one pulmonary tuberculosis patient and one healthy control. Data are 100% complete for BCG, *M*. *tuberculosis* and *S*. *pneumoniae*, 87% for *E*. *coli* and 76% for *C*. *albicans*.

During follow-up, cytokine responses showed a decrease from baseline to day 10 (all but two patients had started on corticosteroids), and an increase again at day 60 and 180 (Supplementary Fig. 4B). The response of TBM patients at day 180 was higher than that of healthy controls for cytokines induced by multiple stimuli: IFN-γ and IL-1RA induced by BCG; TNF-α, IFN-γ, IL-6, IL1-RA induced by *M*. *tuberculosis*; TNF-α, IFN-γ and IL-1RA induced by *S*. *pneumoniae*; and IL-1RA induced by *C*. *albicans* (p < 0.05 for all, data not shown).

### Immune responses correlate to clinical characteristics and survival

Contrary to our hypothesis, we found a broad range in immune responses in TBM patients rather than clearly distinct immune phenotypes (‘high’ and ‘low’ responders). We therefore looked for correlations between cell distribution, cell activation and *ex-vivo* cytokine responses. Myeloid CSF cells correlated with other myeloid cells and lymphoid CSF cells with other lymphoid CSF cells, and the same was true for blood cells. Little or no correlation was seen between the CSF and blood markers (Supplementary Fig. 5).

We next examined the link between *ex-vivo* cytokine responses, blood flow cytometry and clinical characteristics. Activation of CD14^++^CD16^−^ (classical) blood monocytes measured by HLA-DR showed a positive correlation with the first principal component of the *ex-vivo* cytokine response (Spearman rho = 0.52, p = 0.0089), and this was largely driven by the TNF-α, IL-6 and IL-10 responses across the range of stimuli. Not unexpectedly, the first principal component of the *ex-vivo* cytokine response also correlated positively to the following routinely collected clinical parameters: temperature (rho = 0.33, p = 0.0183), blood lymphocytes (rho = 0.40, p = 0.0048) and blood monocytes (rho = 0.34, p = 0.0171).

Finally, we tested if immune markers predicted patient survival. Neither *ex-vivo* cytokine responses nor blood count markers showed an association with outcome, while higher levels of the main CSF lymphoid cell types (αβT and NK cells), were associated with better outcome (Table 3).

**Table 3:** Immune markers as predictor for 180-day mortality.

	CSF			Blood		
	Alive (24)	Dead (16)	p	Alive (23)	Dead (16)	p
*Cell counts*						
CD16^+^ neutrophils	9.9 (2.5–47.8)	12.8 (2.0–36.8)	0.912	5481.7 (3912.4–7092.1)	6563.3 (5421.6–8297.8)	0.209
CD16^low^ neutrophils	0.5 (0.1–3.0)	0.8 (0.0–5.6)	0.890	113.8 (86.8–305.6)	102.1 (42.7–259.3)	0.332
CD14^++^CD16^−^ monocytes	1.4 (0.7–3.5)	0.6 (0.3–2.4)	0.151	300.5 (182.8–438.8)	285.7 (179.7–347.9)	0.511
CD14^++^CD16^+^ monocytes	0.2 (0.1–0.5)	0.2 (0.1–0.4)	0.619	6.8 (2.0–16.3)	7.5 (2.5–12.1)	0.886
CD14^+^CD16^++^ monocytes	0.1 (0.0–0.2)	0.1 (0.0–0.3)	0.740	1.1 (0.5–1.7)	1.4 (0.5–1.6)	0.668
CD56^−^ NK cells	0.9 (0.4–3.6)	0.4 (0.1–1.8)	0.122	6.5 (2.4–9.2)	3.6 (2.7–8.9)	0.568
CD56^+^ NK cells	4.8 (2.0–9.7)	1.2 (0.9–3.6)	0.020	61.2 (35.5–79.8)	46.4 (28.1–96.7)	0.627
CD56^bright^ NK cells	0.7 (0.4-2.0)	0.4 (0.1–0.6)	0.040	2.0 (1.0–3.4)	1.2 (0.8–3.2)	0.361
MAIT cells	0.3 (0.1–0.9)	0.2 (0.0–0.4)	0.082	12.0 (9.5–36.1)	9.6 (6.6–14.7)	0.077
NKT cells	0.0 (0.0–0.1)	0.0 (0.0–0.0)	0.246	0.4 (0.2–0.8)	0.4 (0.1–0.5)	0.511
Vδ2^−^ γδT cells	0.2 (0.2–0.5)	0.2 (0.1–0.4)	0.362	7.3 (2.4–11.2)	5.1 (1.9–11.2)	0.864
Vδ2^+^ γδT cells	0.5 (0.2–1.7)	0.3 (0.1–0.8)	0.258	8.3 (3.0–16.8)	6.2 (3.4–15.2)	1
αβT cells	39.3 (18.5–52.3)	21.1 (14.4–27.8)	0.038	463.8 (182.2–734.0)	284.6 (153.8–447.5)	0.209
*Cell activation*						
CD16^+^ neutrophils (CD69)	4.1 (3.4–6.5)	4.6 (3.5–7.8)	0.531	2.3 (2.0–3.2)	2.5 (2.2–3.1)	0.819
CD16^low^ neutrophils (CD69)	3.2 (2.5–4.0)	3.4 (2.9–4.2)	0.600	3.0 (1.5–19.9)	1.8 (1.3–3.5)	0.493
CD14^++^CD16^−^ monocytes (HLA-DR)	21.2 (12.5–28.5)	21.3 (15.2–38.3)	0.544	9.7 (6.6–12.6)	7.2 (6.4–8.7)	0.346
CD14^++^CD16^+^ monocytes (HLA-DR)	23.6 (13.6–36.8)	26.0 (14.7–34.6)	0.627	17.6 (12.1–27.5)	14.3 (6.8–25.9)	0.530
CD14^+^CD16^++^ monocytes (HLA-DR)	15.6 (6.0–26.0)	6.8 (4.5–10.2)	0.162	8.2 (4.3–15.9)	5.4 (2.0–13.8)	0.248
NK cells (CD69)	9.7 (5.9–17.6)	12.4 (5.0–15.6)	0.785	2.1 (1.4–3.5)	2.0 (1.6–3.5)	0.977
T cells (CD69)	10.9 (7.0–16.4)	11.5 (9.5–18.4)	0.252	1.2 (1.1–2.0)	1.2 (1.1–1.7)	0.822

Presented are CSF and blood cell counts per microliter cell activation as measured by median fluorescence intensity (MFI) of the indicated activation marker. Comparisons are made by Mann-Whitney U for CSF and blood cell counts and activation markers.

Because of the relatively small sample size and skewed distribution, the robust Mann-Whitney U test was chosen. CSF flow cytometry results were missing for one patient and blood flow cytometry results for two patients. One patient was lost to follow-up before day 180.

## Discussion

In this prospective cohort of carefully characterised patients in Indonesia, tuberculous meningitis (TBM) patients showed a strong blood myeloid response compared to healthy controls and pulmonary tuberculosis patients. Within the group of TBM patients, CSF mostly showed a predominance of αβT cells, with highly variable proportions of NK cells and neutrophils, and higher expression of activation markers on CSF monocytes, neutrophils, NK cells and T cells than on these same cell types in blood. Whole blood *ex-vivo* cytokine responses showed a much wider range in TBM patients compared to pulmonary tuberculosis patients and controls. Rather than distinct immune phenotypes, a gradual scale in the immune response was seen with high cytokine responses correlating with other inflammatory markers.

This study is the first to quantify innate cell population concentrations in blood and CSF of TBM patients in comparison to relevant control groups, adding to previous studies that reported low numbers of αβT cells in blood of TBM patients^15^ (probably due to compartmentalisation to CSF^16^), and the presence of γδT and NK cells in CSF^8^. In CSF, besides αβT cells the most abundant cell type were NK cells, an important source of IFN-γ and a critical CSF cytokine in TBM^17^. We also found NKT cells and MAIT cells in CSF, of which the latter have not been described in CSF before. Some patients showed a predominance of neutrophils in CSF, mostly mature, non-apoptotic neutrophils expressing CD16^+^ ^18^. Monocytes were found in low numbers in CSF, with proportionally more CD16^+^ (macrophage-like) subsets than in blood and with high expression of the activation marker HLA-DR. Leukocyte activation was much higher in CSF than blood, with very little correlation between compartments, indicating that future studies cannot rely on blood flow cytometry or *ex-vivo* stimulation assays when aiming to characterise the cerebral immune response.

Whole blood cytokine responses showed a much wider range in TBM patients compared to the two control groups, ranging from non-responsiveness to hyper-responsiveness. This gradient in production of TNF-α, IL-1β, IL-6 and IL-10 was seen in response to the mycobacterial stimuli BCG and *M*. *tuberculosis* as well as to non-mycobacterial stimuli *S*. *pneumonia* and *C*. *albicans*. *Ex-vivo* cytokine responses showed a positive correlation with blood lymphocyte and monocyte counts and especially with HLA-DR expression on monocytes. *Ex-vivo* cytokine responses also correlated positively with body temperature, which predicted early mortality in our previous study on routine inflammatory markers in 499 HIV-negative TBM patients^5^. Other studies have shown that immunoparalytic sepsis patients with low cytokine responses have higher mortality later during the course of disease, possibly because of a higher rate of secondary infection^19^, but we could not confirm this in our present study.

At 10 days post admission, a remarkable decrease in CSF myeloid cells and all lymphoid cells except αβT cells was seen. Different from a Vietnamese study analysing dexamethasone versus placebo that used comparable methodology^8^, *ex-vivo* cytokine responses were strongly down regulated in our patients at day 10. We consider this to be the effect of corticosteroid treatment because the cytokine production was restored after cessation of steroids after 6 weeks (day 60 and 180 measurements). At day 180, proinflammatory cytokine production were even higher than in healthy controls, both in response to mycobacterial stimuli as shown before^20^, as well as to unrelated stimuli *C*. *albicans* and *S*. *pneumoniae*. Our findings show similarities to HIV-associated cryptococcal meningitis with proportionally overrepresented CD56^bright^ NK cells and CD14^++^CD16^+^ and CD14^+^CD16^++^ monocytes in CSF compared to blood^21^ and a subgroup of patients with low HLA-DR on monocytes and low *ex-vivo* TNF-α^22^.

Our study has several limitations. Although much larger than previous studies, caution is warranted in its interpretation because of the many comparisons relative to the number of included individuals. Our follow-up was 99% complete, but high early mortality compromised follow-up sampling. Also, CSF leukocytes, especially myeloid cells, show an almost 40% loss from 30 to 60 minutes after lumbar puncture^23^. To limit cell loss, we immediately resuspended CSF cells in culture medium. For the *ex-vivo* cytokine assay, we chose 24 hours incubation. This is optimal for measuring for monocyte-derived cytokines^24^, but relatively short for IFN-γ. Cytokines in CSF were not measured in this study. The correlations of the *ex-vivo* cytokine response, require validation in a separate cohort given their number.

What is the clinical relevance of our findings? Besides better supportive care^25^ and higher dose rifampicin^26^, immunomodulatory therapy may be a third strategy to improve treatment of TBM. In the sepsis field a one-size-fits-all approach has not been successful^27^. Our findings suggest that the same may be true for TBM. There could be a role additional anti-inflammatory therapy, i.e. with aspirin^4,28^, or interleukin-1 receptor antagonist for patients with a strong inflammatory response. At the same time, patients with a low response might have a better outcome without corticosteroid treatment, or might even benefit from adjuvant IFN-γ therapy to boost their immune response, as has been done successfully in a child with protracted TBM^29^. Preferably, future host-directed trials should include immune markers to allow for post-hoc identification of subgroups benefitting from the initiated therapy. Good candidates are blood monocyte HLD-DR expression, well generalizable to other settings, and *ex-vivo* cytokine responses. Because of the lack of correlation between blood and CSF compartments, we recommend to also include CSF markers when studying adjuvant therapies.

In conclusion, TBM patients show a previously not appreciated strong myeloid blood response, a remarkably broad lymphoid CSF response including innate lymphocytes, and an unexpectedly large gradient in the immune response, with little correlation between blood and CSF compartments. We recommend integrating the assessment of the immune response in future randomised clinical trials evaluating host-directed therapy in TBM.

## Electronic supplementary material

Supplementary figures and table

## Data Availability

The datasets generated in the current study are available from the corresponding author on reasonable request.
