# Supplementary material for: Immune cell characteristics and cytokine responses in adult HIV-negative tuberculous meningitis: an observational cohort study
Source: Sci Rep. 2019 Jan 29;9:884. doi: 10.1038/s41598-018-36696-3 (PMC6351535; doi:10.1038/s41598-018-36696-3)
Supplement: Supplementary file 1 — Supplementary figures and table [file 41598_2018_36696_MOESM1_ESM.pdf]

Supplementary figures to

# *"Immune cell characteristics and cytokine responses in adult HIV-negative tuberculous meningitis: an observational cohort study"*

Arjan van Laarhoven, Sofiati Dian, Suzanne van Dorp, Feby Purnama, Valerie ACM Koeken, Emira Diandini, Fitria Utami, Resvi Livia, Lika Apriani, Edwin Ardiansyah, Rob ter Horst, Mihai G Netea, Tri Hanggono Achmad, Philip C Hill, Rovina Ruslami, Bachti Alisjahbana, James E Ussher, Agnes Indrati, Ayesha Verrall, Ahmad Rizal Ganiem, Reinout van Crevel

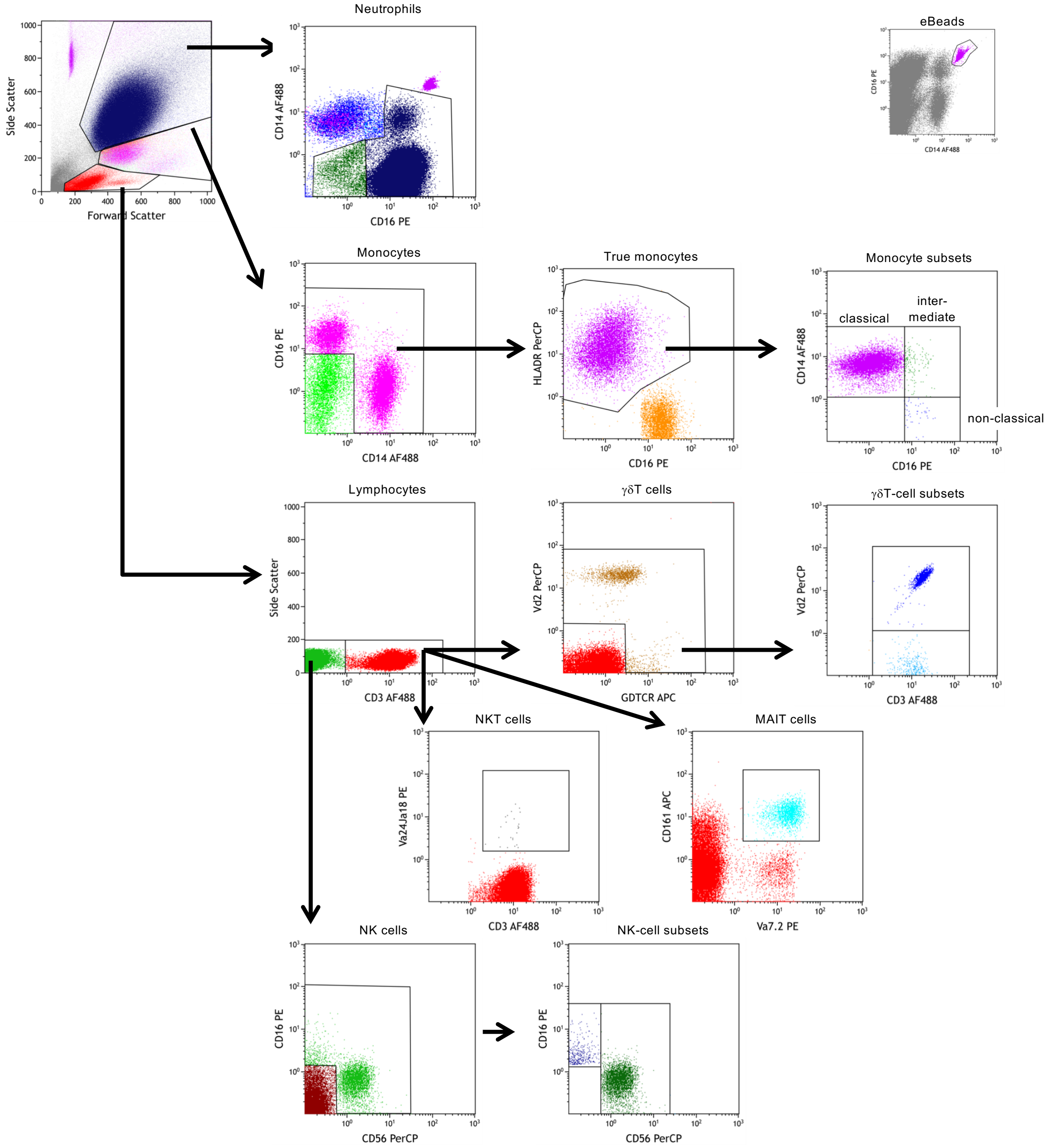


**Supplementary figure 1** (A) flow cytometry gating strategy. αβT cells are calculated by subtracting NKT cells, γδT cells and MAIT cells from the total number of CD3⁺ lymphocytes.

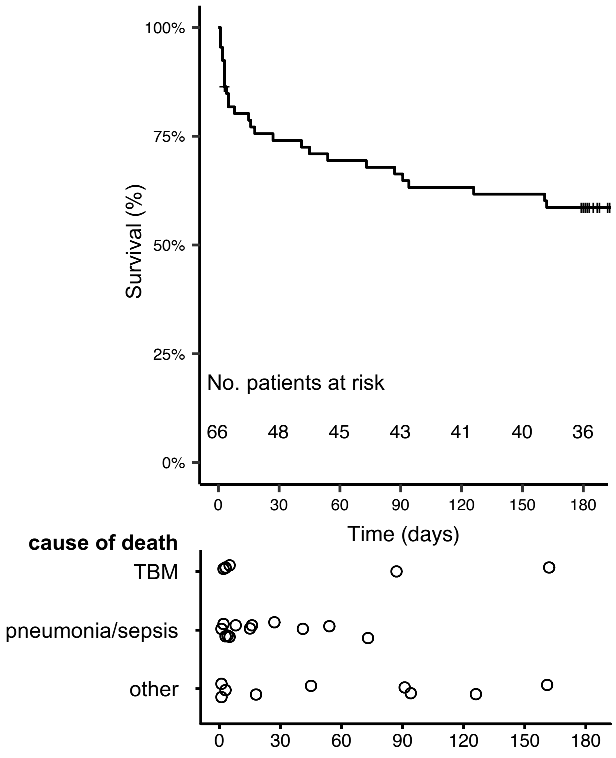


(B) Mortality of tuberculous meningitis patients in the first 180 days of follow-up. Outcome data were missing for one patient. Presumptive cause of death is depicted below the Kaplan-Meier graph in three categories: 1) tuberculous meningitis-related causes: 4 because of increased intracranial pressure, 2 because of a paradoxical reaction, 2) pneumonia (8) or sepsis (4), and 3) miscellaneous: 3 aspiration pneumonia, 3 suspected cardiac arrest or pulmonary embolism, 1 head injury, 1 metabolic encephalopathy and 1 with unknown cause.

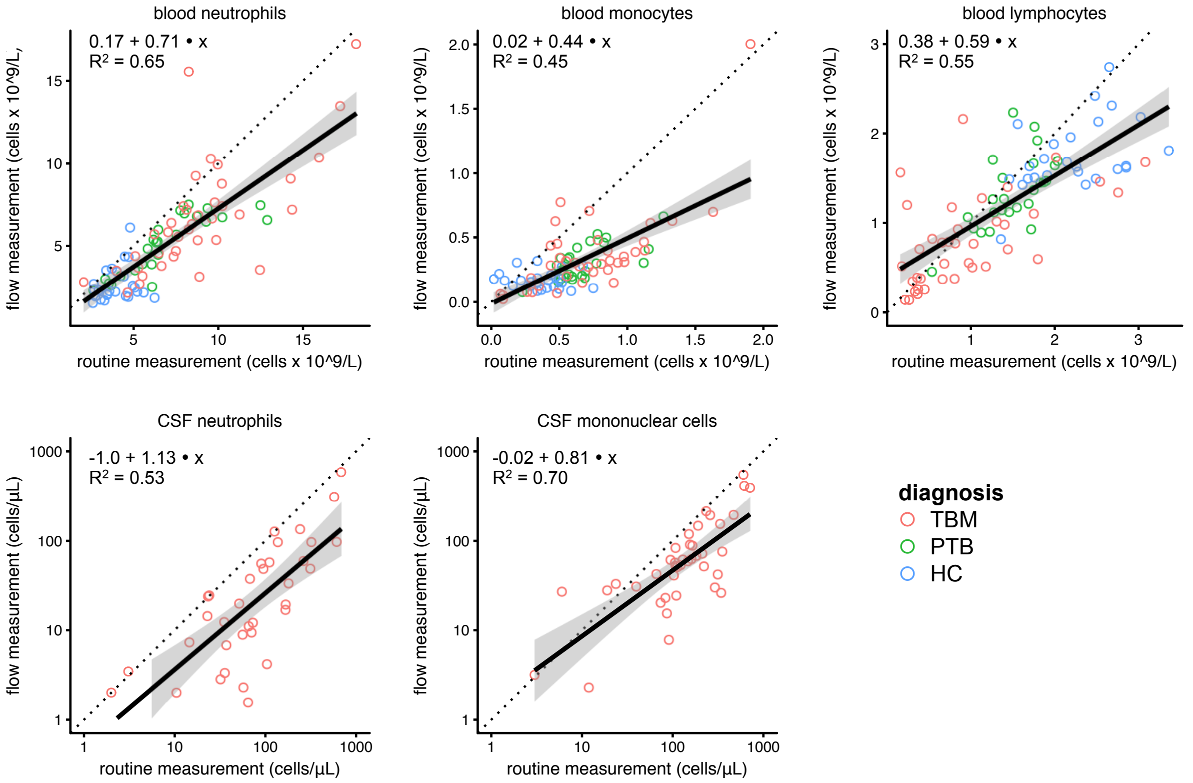


(C) Regression lines with 95% confidence intervals for the correlation between cell counts as measured routinely (x-axis), and with flow cytometry (y-axis) for blood and CSF. Regression formulas and regression coefficients ($R^2$) are printed on the graphs. Note: CSF cells are plotted on a $^{10}$log-axis and regression coefficients are $^{10}$log-transformed.

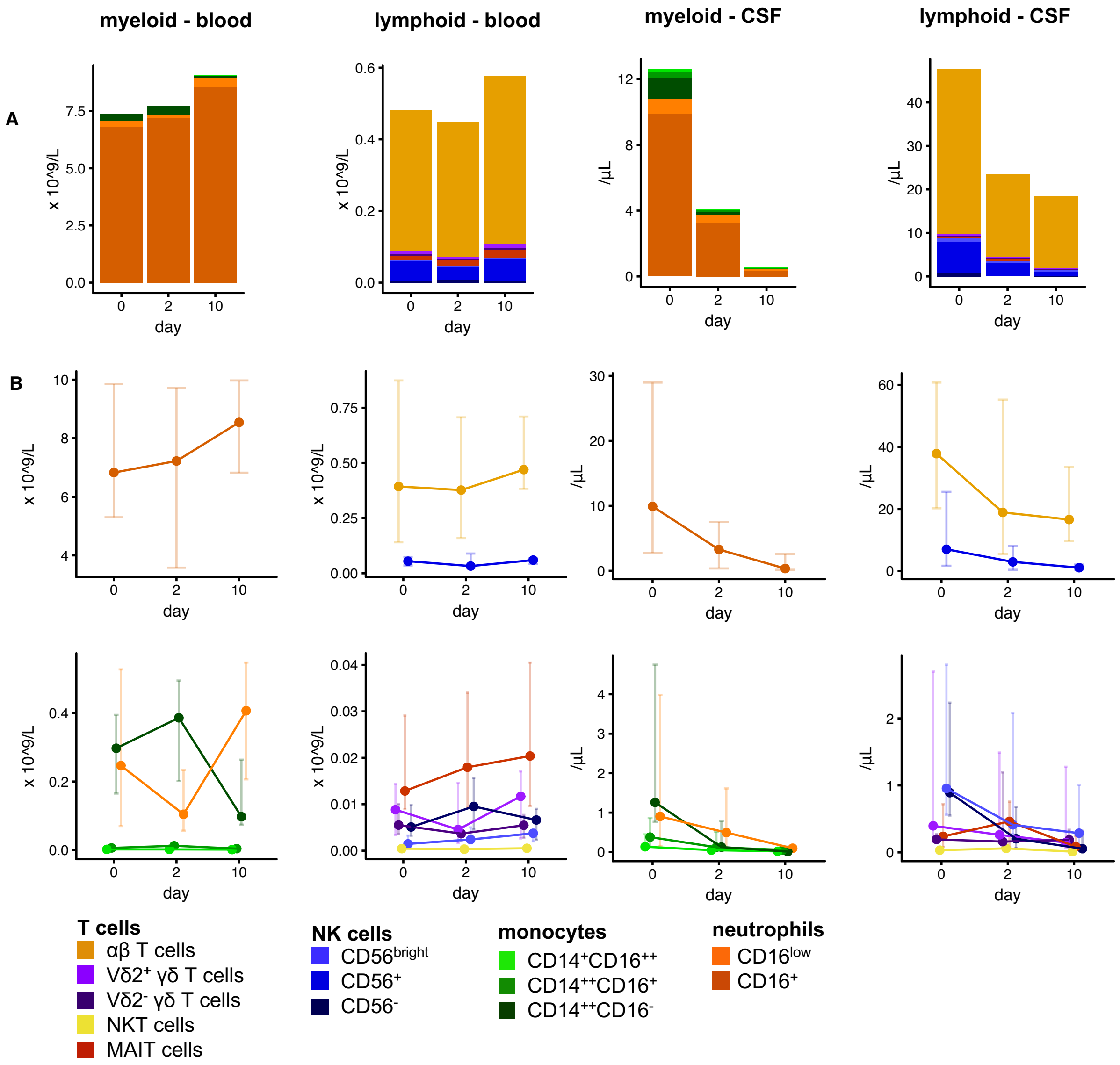


**Supplementary figure 2** Flow cytometry follow-up study results of tuberculous meningitis patients who survived past day 10 (n = 15). Results show median (A) or median with interquartile range (B) for day 0, 2 and 10 for myeloid and lymphoid cell types separately in blood (left) and CSF (right). Note the different y-axis limits. Data is ≥ 80% complete for all time-points for blood and is ≥ 87% complete for CSF.

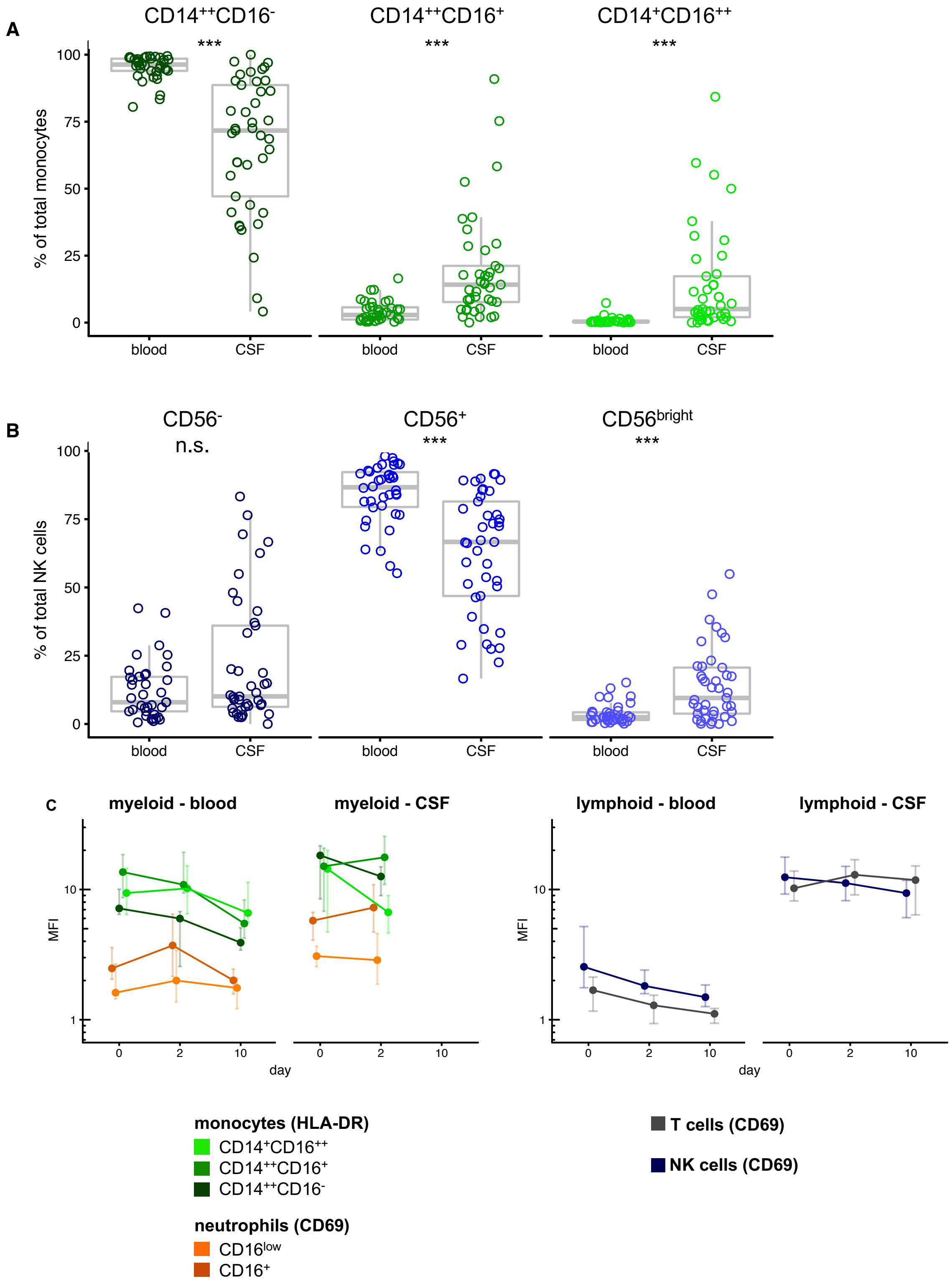


**Supplementary figure 3** Flow cytometry results of proportions of monocyte (A) and NK cell (B) subtypes in blood versus CSF of tuberculous meningitis patients. *** p<0.001. (C) Activation marker expression by indicated cell populations during patient follow-up, all of whom survived past day 10 (n = 15). Data is ≥ 80% complete for all time-points for CSF and blood. Note: day 10 myeloid activation markers in CSF could not reliably be measured because of the low cell counts.

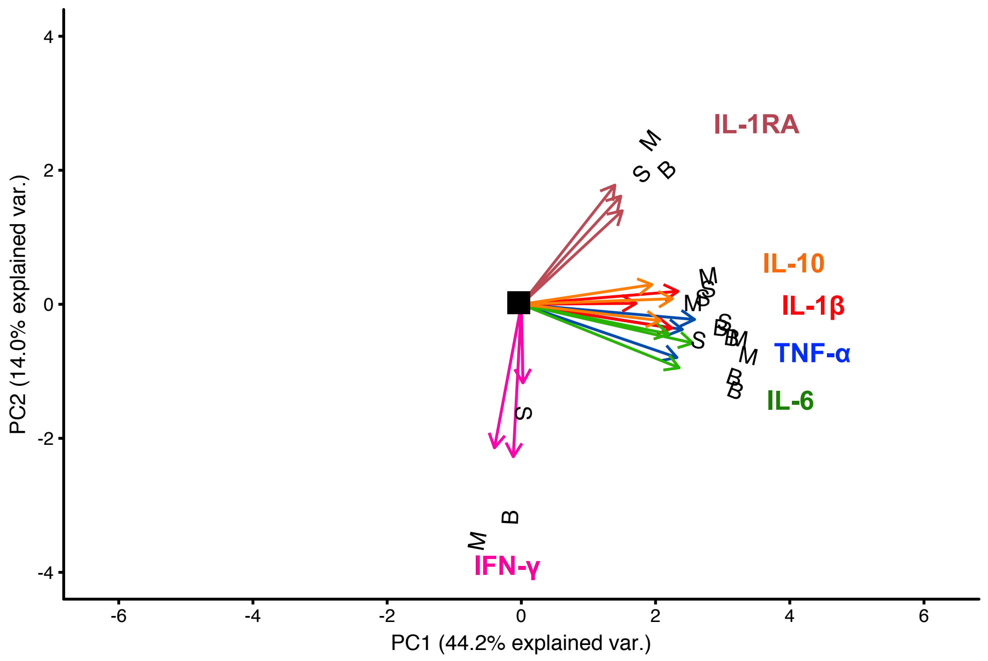


**Supplementary figure 4** (A) Loadings for principal component analysis on ex-vivo cytokine data. B = BCG, M = *M. tuberculosis* and S = *S. pneumoniae*.

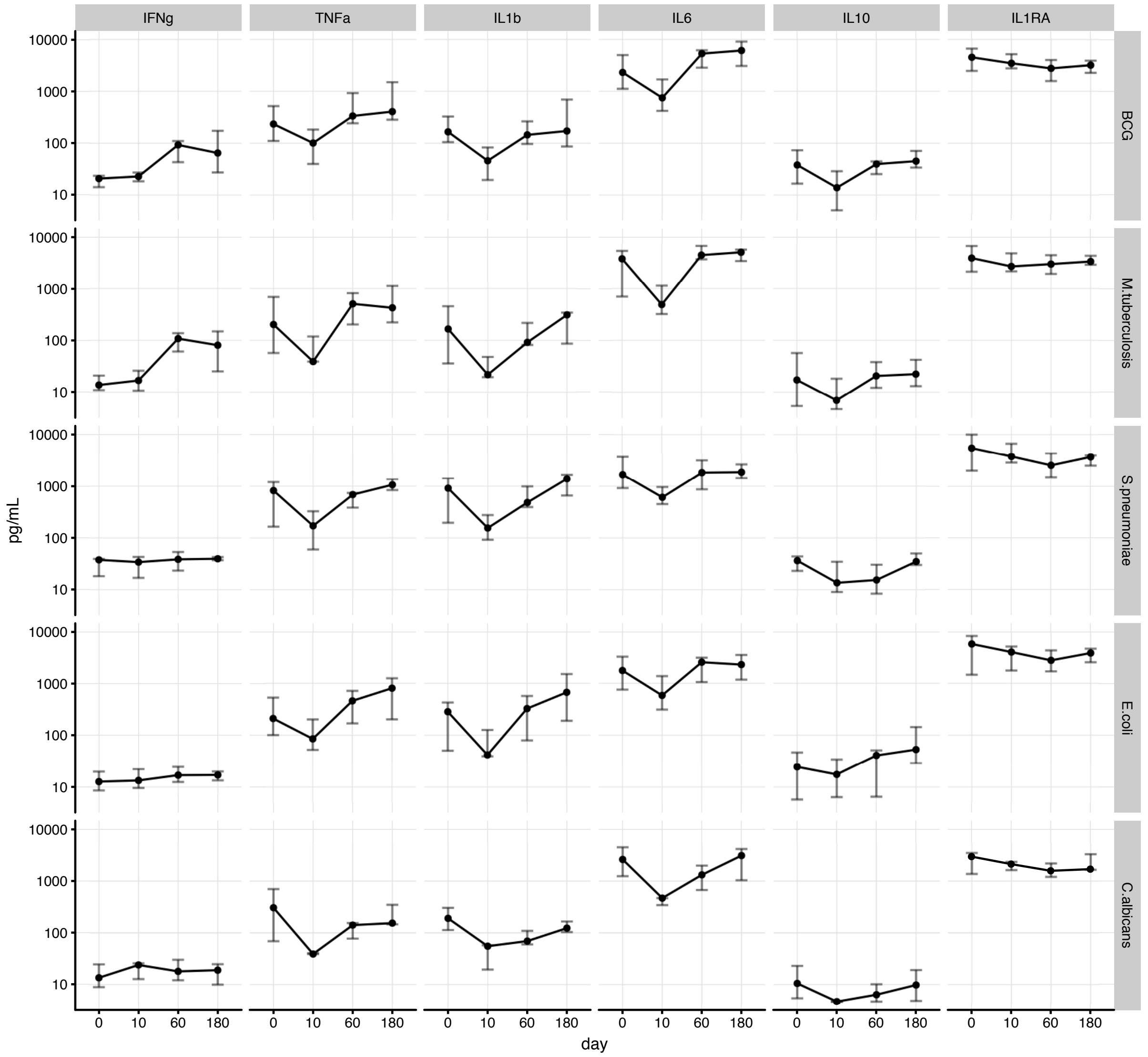


(B) Ex-vivo whole blood cytokine results for tuberculous meningitis patients in the follow-up study who survived past day 180 (n = 15). Results show median with interquartile range for all five stimuli (ordered vertically) for all six measured cytokines (horizontally). Patients were selected who had complete data at day 0 and missed maximally one of the follow-up time-points. Data is 93% complete for day 10, 73% for day 60 and 53% for day 180. Missing data were due to technical errors.

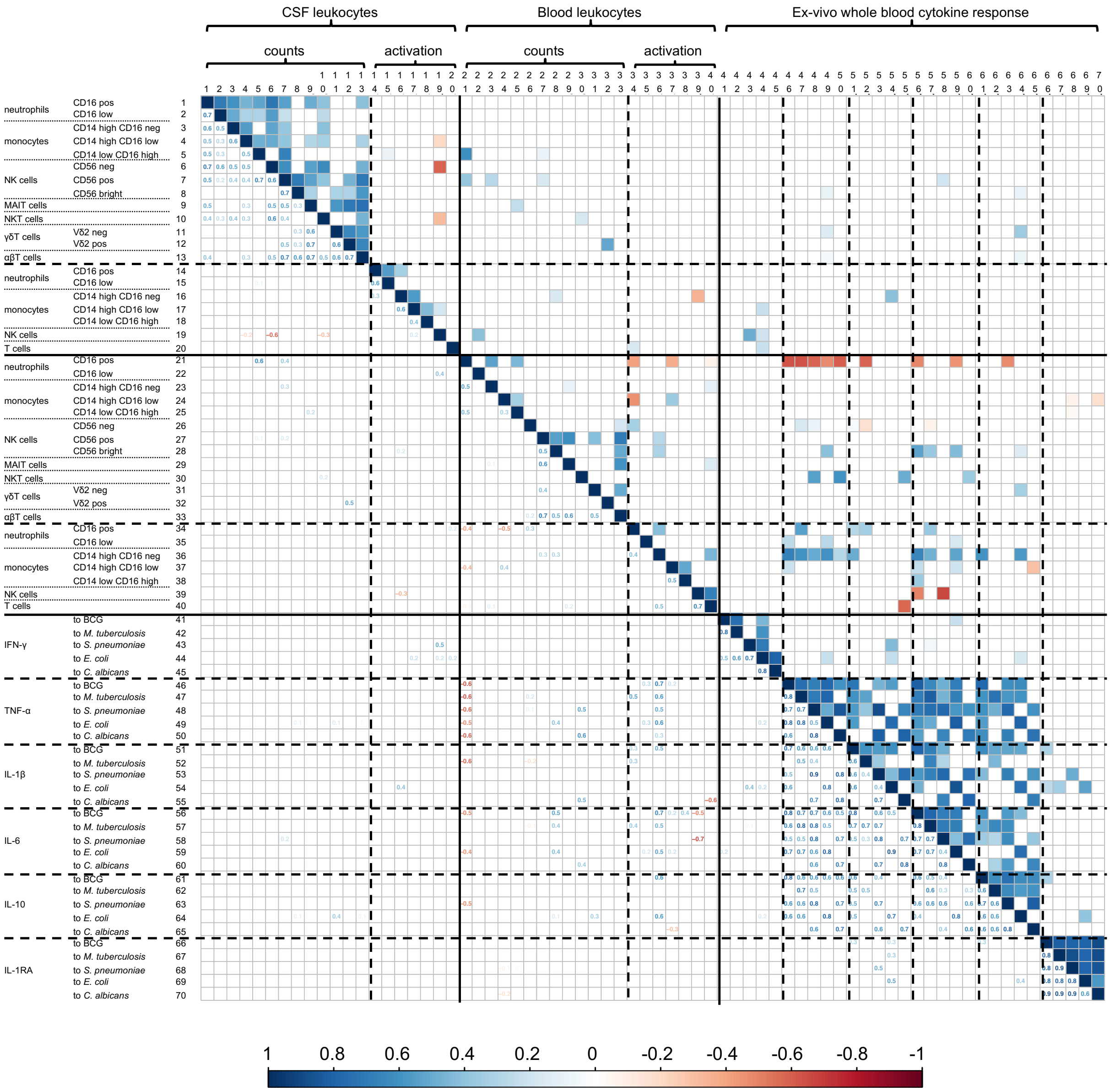


**Supplementary figure 5** Correlation matrix for tuberculous meningitis patient CSF and blood cell counts and activation markers, as well as blood ex-vivo whole blood response using Spearman correlation. Pair-wise complete data was included.

# Supplementary table

Supplementary table 1: Clinical, cerebrospinal fluid and blood parameters of tuberculous meningitis patients

| Variable | n/N or median | % or IQR |
|---|---|---|
| **Clinical features** | | |
| Sex | 37/67 | (56%) |
| Age | 27 | (21–38) |
| Duration of main complaint | 4 | (2–7) |
| Duration of the first neurological symptom | 14 | (7–21) |
| Tuberculous meningitis grade | | |
| I | 2/67 | (3%) |
| II | 58/67 | (87%) |
| III | 7/67 | (10%) |
| Temperature, °C | 37.8 | (37.0–38.2) |
| Glasgow Coma Scale score | 13 | (11–14) |
| Seizures present | 1/66 | (2%) |
| Motor abnormalities present | 38/66 | (57%) |
| Cranial nerve palsy present | 51/66 | (6%) |
| Abnormal chest radiograph findings | 50/67 | (75%) |
| **Cerebrospinal fluid** | | |
| CSF to blood glucose ratio | 0.14 | (0.09–0.20) |
| Protein, mg/dL | 221 | (150–386) |
| Culture, % positive | 46/67 | (69%) |
| **Blood** | | |
| Haemoglobin, g/dL | 12.5 | (10.5–13.8) |
| Thrombocytes, x $10^9$/L | 298 | (234–379) |
| **Outcomes** | | |
| Length of hospital stay in days | 21 | (12,23) |
| Alive at discharge | 51/66 | (76%) |
| Outcome at day 180 | | |
| Alive | 39/71 | (58%) |
| Deceased | 27/67 | (40%) |
| Lost to follow-up | 1/67 | (2%) |

Continuous variables are presented as median with interquartile range and categorical variables as number and proportion.

IQR = interquartile range. Additional routine inflammatory markers can be found in Table 1.
